# Immune cell profiling supports early prediction of sepsis-associated acute kidney disease using a decision tree algorithm

**DOI:** 10.1186/s40364-025-00870-3

**Published:** 2025-12-30

**Authors:** Mei-Yi Wu, Chun-Hao Lai, Yen-Ling Chiu, Po-Chun Tseng, Josephine Diony Nanda, Chiou-Feng Lin, Mai-Szu Wu

**Affiliations:** 1 Division of Nephrology, Department of Internal Medicine, Taipei Medical University-Shuang Ho Hospital, New Taipei City, 23561 Taiwan; 2 Division of Nephrology, Department of Internal Medicine, School of Medicine, College of Medicine, Taipei Medical University, Taipei, 11031 Taiwan; 3 TMU Research Center of Urology and Kidney, Taipei Medical University, Taipei, 11031 Taiwan; 4 Institute of Epidemiology and Preventive Medicine, College of Public Health, National Taiwan University, Taipei, 10055 Taiwan; 5 Department of Microbiology and Immunology, School of Medicine, College of Medicine, Taipei Medical University, No. 250, Wu-Xing St., Taipei, 11031 Taiwan; 6 Division of Nephrology, Department of Medicine, Far Eastern Memorial Hospital, New Taipei City, 220216 Taiwan; 7 Department of Medical Research, Far Eastern Memorial Hospital, No. 121, Sec. 2, Nanya S. Rd., Banqiao Dist., New Taipei City, 220216 Taiwan; 8 Graduate Institute of Medicine and Graduate Program in Biomedical Informatics, Yuan Ze University, Taoyuan, 320315 Taiwan; 9 Graduate Institute of Clinical Medicine, College of Medicine, National Taiwan University, Taipei, 100233 Taiwan; 10 Core Laboratory of Immune Monitoring, Office of Research & Development, Taipei Medical University, Taipei, 11031 Taiwan; 11 Department of Parasitology, Faculty of Medicine, Public Health and Nursing, Universitas Gadjah Mada, Yogyakarta, Indonesia; 12 Graduate Institute of Medical Sciences, College of Medicine, Taipei Medical University, Taipei, 11031 Taiwan

**Keywords:** Sepsis, Acute kidney disease, Immune cell profiling, Machine learning, Decision tree

## Abstract

Sepsis is a major cause of acute kidney injury, progressing to sepsis-associated acute kidney disease (SA-AKD). This study explores SA-AKD prediction by combining immune cell profiling. Peripheral immune cell expression and phenotypes were analyzed in sepsis patients without (*n* = 97) and with *(n* = 41) SA-AKD, admitted to a hospital (2020–2022). Blood urea nitrogen and creatinine levels were measured, and a decision tree (DT)-based model was used to evaluate their predictive power in the training (*n* = 106) and validation (*n* = 32) cohorts. The DT model, incorporating naïve Treg and CD56^dim^ NK cells along with clinical parameters, showed high accuracy in predicting SA-AKD. The model using blood urea nitrogen as the first node reached 89.62% accuracy (sensitivity: 94.4% and specificity: 87.14%; area under the curve = 0.91). The model starting with creatinine showed 89.62% accuracy. Validation results confirmed an 81.25% accuracy. Profiling specific immune cells may enable pre-evaluation of SA-AKD.

**Supplementary Information:**

The online version contains supplementary material available at 10.1186/s40364-025-00870-3.

**To the Editor,**

Sepsis is a leading cause of acute kidney injury (AKI), a condition with a high risk of progression to acute kidney disease (AKD) and chronic kidney disease (CKD), contributing to poor prognosis and increased mortality among critically ill patients [1–3]. Despite advances in clinical management, early prediction of sepsis-associated AKD (SA-AKD) remains challenging due to limitations in conventional biomarkers such as blood urea nitrogen and creatinine. Recent findings suggest that immune dysregulation during sepsis plays a key role in renal injury [4–6], yet the transition from AKI to AKD remains poorly understood.

We conducted a prospective observational study to investigate whether peripheral immune cell profiling, in combination with machine learning (ML), could facilitate early SA-AKD prediction. Blood samples were collected from 138 sepsis patients, including 41 with SA-AKD (Table S1; Fig. S1A), and further segmented into a training group (*n* = 106) and a validation group (*n* = 32). Clinical laboratory values (Table S2) and 55 immune cell subsets were analyzed (Tables S3, S4). On Day 7 post-AKI diagnosis, patients who progressed to SA-AKD exhibited significant increases in blood urea nitrogen and creatinine (Figs. S1B, S2A), along with neutrophilia and lymphopenia on CBC (Figs. S1C, S2B). Flow cytometric profiling of peripheral blood mononuclear cells (Fig. S3A) revealed that SA-AKD patients had reduced naïve Th (CD62L^+^CD4^+^CD3^+^CD56^−^), naïve Th1 (CD3^+^CD4^+^CXCR3^+^CD45RA^+^), and CD56^bright^ NK (CD3^−^CD56^bright^ CD16^−^) cells, but elevated CD56^dim^ NK (CD3^−^CD56^dim^ CD16^−^) cells (Figs. S3B, C, S4A, B). The results of principal component analysis on these immune subsets revealed significant differences with a threshold score of 0.508, resulting in prediction accuracies of 75.5% in the training group and 59.4% in the validation group (Fig. S3D). The overall prediction accuracy was 67.5% (Fig. S4C). However, the sensitivity and specificity were modest (Figs. S3E, S4D). These findings suggest the potential of immune cell profiling for pre-evaluating the immune risk of SA-AKD, although further refinement of such profiling is required.

We next developed and evaluated a decision tree (DT)-based ML model integrating immune cell markers. Among various algorithms tested, the DT model, but not the support vector machines (SVM), K-nearest neighbors (KNN), showed superior performance (accuracy: 85%) (Table 1). Using CD56^dim^ NK and naïve Treg cells (CD3^+^CD4^+^CD25^+^CCR5^+^CD45RA^+^) as initial decision nodes (Fig. S5A), the DT model achieved 84.91% accuracy in the training cohort and 81.25% in the validation cohort (Figs. S5B, S6A). Predictive accuracy using standard renal markers alone was lower (77.36% accuracy in the training cohort and 75% in the validation cohort) (Figs. S7, S6B). Importantly, combining renal indices with immune cell profiling significantly improved model performance. A DT model incorporating blood urea nitrogen or creatinine as the first node, followed by naïve Treg and CD56^dim^ NK cells, yielded an accuracy of 89.62% (AUC = 0.91–0.92) (Fig. 1A–B). Validation data confirmed the robustness of this model (accuracy: 81.25%; AUC = 0.89). Additionally, integrating all four parameters into a composite DT model retained high accuracy in both training and validation cohorts (Fig. S8). Mechanistically, decreased naïve Tregs correlated negatively, while increased CD56^dim^ NK cells positively, with markers of renal injury (Fig. 1C–D; Fig. S9), suggesting functional relevance in disease progression.

**Fig. 1:**
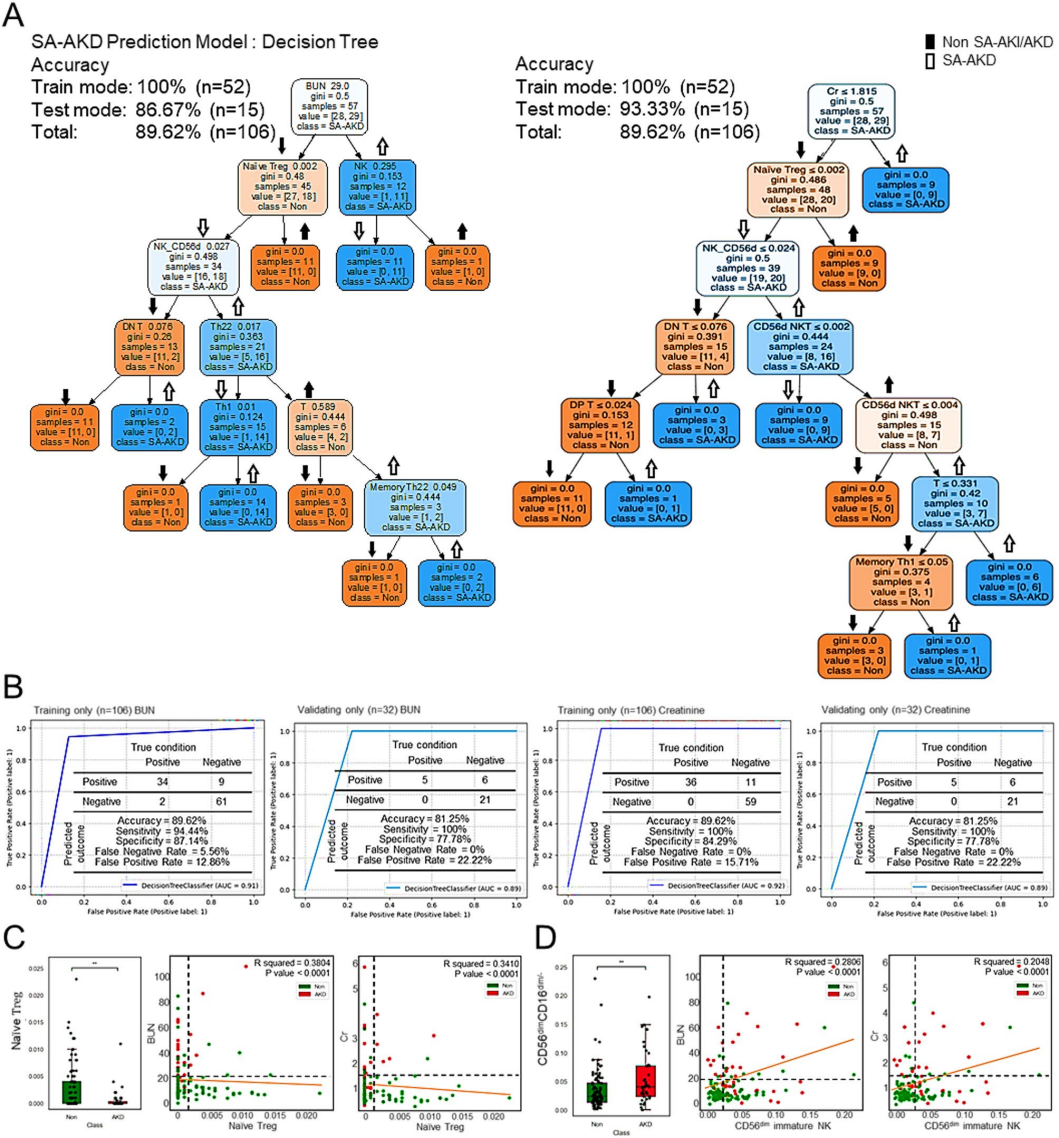
Decision tree–based immune risk prediction for sepsis-associated acute kidney disease (SA-AKD). (**A**) Accuracy of SA-AKD predictions made using DT. (**B**) Sensitivity, specificity, and AUC of SA-AKD predictions made using DT. On the basis of the results of DT predictions, the expression of (**C**) naïve Treg cells and (**D**) CD56dim NK cells was measured in patients without SA-AKD and with SA-AKD. Correlation of the expression of these cells with renal function parameters, specifically with blood urea nitrogen (BUN) and creatinine (Cr) levels. Significant differences were assessed using the independent-samples Kruskal-Wallis test. ∗∗*P* < 0.01. ns, not significant

**Table 1:** Accuracy of various machine learning models in predicting the risks of sepsis-associated acute kidney disease (SA-AKD) progression

Classification	Cell population	Train/Test 80%/20%	Test data accuracy (*n* = 106)		
			SVM	KNN	DT
Non SA-AKD vs. SA-AKD	55	52/15	70%	50%	85%

DT, decision tree; KNN, K-nearest neighbors; SA-AKD, sepsis-associated acute kidney disease; SVM, support vector machines

Furthermore, the integrated DT model combining renal indices (BUN and Cr) with immune cell subsets (naïve Treg and CD56^dim^ NK cells) achieved robust predictive performance. It showed an accuracy of 88.7%, AUC of 0.91, and balanced metrics including sensitivity 94.4%, specificity 85.7%, PPV 77.3%, NPV 96.8%, and F1-score 85.0% (Table S5), supporting its reliability and discriminative power. Additionally, we performed 5-fold cross-validation for internal validation and model stability assessment. The results demonstrate that, after class balancing, the SA-AKD classification model achieved stable and reliable predictive performance. The balanced accuracy, high sensitivity, and specificity (> 80%) indicate robust generalization and effective discrimination between SA-AKD and non-AKD cases (Table S6), supporting the model’s validity for preliminary clinical prediction despite a limited sample size. To enhance the evaluation of model robustness, we incorporated SHAP value analysis to interpret feature contributions within the SA-AKD classification model (Fig. S10). The SHAP summary plot revealed that BUN, Cr, and CD56^dim^ NK cells exhibited the highest SHAP values, underscoring their dominant influence on predictive outcomes. The consistent feature ranking across folds supports the stability and interpretability of the model, confirming that renal indices and immune cell subsets jointly drive accurate SA-AKD prediction.

Our approach demonstrates that immune cell profiling, combined with ML, offers significant advantages over conventional diagnostics in the early detection of SA-AKD. Notably, some patients with normal renal indices at admission progressed to SA-AKD, underscoring the inadequacy of relying solely on biochemical markers. The addition of immune cell-based features improved sensitivity and reduced false-negative rates, enabling timely risk stratification and intervention. Our study used high-dimensional flow cytometry to specify changes in lymphocyte subsets. The identification of CD56^dim^ NK cells and naïve Tregs as key predictors of SA-AKD aligns with current immunopathological insights. Decreased naïve Th and Th1 cells likely reflect immunosuppression or lymphocyte apoptosis, while increased CD56^dim^ NK cells suggest innate immune overactivation. These observations align with prior studies identifying NK cells as critical mediators in sepsis-related organ dysfunction [7, 8]. CD56^dim^ NK cells, the primary cytotoxic subset, contribute to tubular and endothelial injury via perforin, granzymes, and IFN-γ, promoting microvascular inflammation and cytokine amplification [7]. Conversely, naïve Tregs possess limited suppressive capacity, and their impaired differentiation during sepsis aggravates immune dysregulation and renal injury [9]. Together, these reciprocal changes reflect an imbalance between cytotoxic and regulatory immunity that drives SA-AKD progression, consistent with emerging evidence of immune exhaustion and cytokine-driven renal pathology [10]. Despite promising results, our study had limitations. The limited sample size hindered broader algorithm validation. While the small validation cohort reduces statistical power and external reliability, larger multi-center studies to confirm our findings are therefore suggested. Furthermore, it is necessary to use appropriate controls, such as non-sepsis AKI patients or healthy individuals, to assess disease specificity sufficiently. Finally, the functional roles of naïve Treg and CD56^dim^ NK cells in SA-AKD pathophysiology remain to be elucidated experimentally.

Recent ML frameworks have increasingly integrated such immune parameters for outcome prediction in sepsis and AKI, but not AKD, outperforming conventional scoring systems. Explainable ML models, including SHAP-based XGBoost and LightGBM, have identified immune and metabolic predictors of persistent SA-AKI and mortality [11–13], while deep learning approaches incorporating multiomic immune features further refine patient stratification and reveal immunoregulatory targets [14]. Collectively, the opposing dynamics of NK expansion and Treg insufficiency revealed by our model exemplify a dysregulated innate–adaptive immune axis that ML approaches increasingly capture in sepsis-related kidney dysfunction, underscoring the biological and translational validity of our immune-based framework.

In conclusion, we present a novel immune-based DT algorithm that enables early and accurate prediction of SA-AKD in septic patients. While high-parameter flow cytometry was used only for discovery, our findings suggest that simplified immune panels capturing key biomarkers could be developed for routine clinical use and broader accessibility. Integrating immune cell profiling with clinical parameters significantly enhances diagnostic precision, offering a practical tool for early therapeutic decision-making in critical care settings.

## Supplementary Information

Below is the link to the electronic supplementary material.

Supplementary Material 1

## Data Availability

No datasets were generated or analyzed during the current study.

## Abbreviations

AKD: Acute kidney disease
CKD: Chronic kidney disease
DT: Decision tree
KNN: K-nearest neighbors
NK: Natural killer cell
PBMC: Peripheral blood mononuclear cell
Treg: Regulatory T cell
SA-AKD: Sepsis-associated acute kidney disease
SVM: Support vector machines
