# Supplementary material for: Immune cell profiling supports early prediction of sepsis-associated acute kidney disease using a decision tree algorithm
Source: Biomark Res. 2025 Dec 30;13:160. doi: 10.1186/s40364-025-00870-3 (PMC12754851; doi:10.1186/s40364-025-00870-3)

**Supplemental Material**

**Methods**

**Study Design**

Patient recruitment was conducted at Shuang-Ho Hospital, Ministry of Health and Welfare. This is a single-center study with prospective follow-up. From 2020 to 2022, blood samples were collected from 138 patients with sepsis in the university-affiliated hospital. Regular laboratory examinations, including complete blood count (CBC) tests and renal function serology, were performed to monitor kidney injuries. The exclusion criteria include participants younger than 20 years old, cancer patients, pregnant women, and those who have undergone kidney transplantation, continuous renal replacement therapy or surgery in the past 3 months. Blood samples were collected on the day after obtaining informed consent from the participants, and peripheral blood mononuclear cells (PBMCs) were isolated from the blood sample. We used this data on day 1 to predict whether septic AKI patients would develop AKD on day 7. Participants were segmented on the basis of clinical AKD criteria into non-SA-AKD (*n* = 97) and SA-AKD (*n* = 41) groups and further segmented into a training group (2020-2021, *n* = 106) and a validation group (2022, *n* = 32).

**Laboratory Examination**

Serum creatine and blood urea nitrogen tests are commonly used to assess kidney function in individuals with sepsis and potential kidney injuries. In the present study, sepsis is an overwhelming reaction to infection that comes with high morbidity and mortality. All tests were conducted at the university-affiliated hospital in accordance with global laboratory accreditation standards, including the International Organization for Standardization accreditation standard 15189 and the College of American Pathologists Survey accreditation.

**Immune Cell Profiling**

Peripheral blood mononuclear cells (PBMCs) were purified and analyzed for immune cell subpopulations using multiparameter flow cytometry (Attune NxT Flow Cytometer, Invitrogen) equipped with a three-laser, 11-color detection system. Cells were first incubated with an Fcγ receptor–blocking reagent for 30 minutes at room temperature in the dark, followed by surface staining with fluorescence-conjugated antibodies. Panel #1 included antibodies specific for CD3, CD4, CD8, CD11c, CD14, CD16, CD19, CD25, CD56, CD62L, and HLA-DR, whereas Panel #2 included antibodies against CD3, CD4, CXCR3, CCR3, CCR5, CCR6, CCR10, CXCR5, CD45RA, and CD45RO (Table S7). Unstained cells were used as negative controls, and Attune Performance Tracking Beads were employed to ensure instrument accuracy and sensitivity, allowing easy identification of potential shifts or trends in performance.

Data acquisition and analysis were performed using Attune NxT Software. Side scatter and forward scatter parameters were used to gate and isolate mononuclear cells and granulocytes. In Panels #1–2, a multicolor gating strategy was applied to identify 55 major immune cell subsets and T helper (Th) subpopulations from PBMCs. In Panel #1, PBMCs were first gated to define major lymphocyte lineages. CD3 and CD19 expression distinguished T cells (CD3⁺CD19⁻) and B cells (CD3⁻CD19⁺). CD3 and CD56 expression identified natural killer T (NKT) cells (CD3⁺CD56⁺) and natural killer (NK) cells (CD3⁻CD56⁺). Within the T-cell compartment, CD4 and CD8 gating defined CD4⁺ T helper (Th) and CD8⁺ cytotoxic T (Tc) subsets, as well as CD4⁺ NKT and CD8⁺ NKT populations. CD4⁺CD25⁺ cells were identified as regulatory Th (Treg) cells, and CD62L and HLA-DR expression further discriminated activated, naïve, and memory Th subsets. Similarly, CD8 and CD25 expression identified regulatory Tc cells, while CD62L and HLA-DR distinguished activated, naïve, and memory Tc populations. CD14 and CD16 were used to delineate monocyte subsets, including classical (CD14⁺⁺CD16⁻), intermediate (CD14⁺⁺CD16⁺), and non-classical (CD14⁺CD16⁺⁺) monocytes. CD11c and HLA-DR expression defined immature and mature dendritic cells (DCs). In Panel #2, CD3⁺CD4⁺ T cells were further analyzed to define functional Th subpopulations. CXCR3 and CCR3 expression distinguished Th1 and Th2 subsets, respectively. CCR6 and CCR10 expression identified Th17 (CCR6⁺CCR10⁻) and Th22 (CCR10⁺CCR6⁺) cells. CXCR5⁺CCR10⁻ cells were classified as T follicular helper (Tfh) cells, whereas CD25⁺CCR5⁺ cells represented regulatory T (Treg) cells. Each Th subset was further categorized into naïve and memory phenotypes based on CD45RA and CD45RO expression.

As described in our previous studies, all values are expressed as the percentage of total PBMCs, representing relative proportions rather than absolute counts. This normalization allows for direct comparison between samples and experimental runs, ensuring data reproducibility across different flow cytometry sessions. For standardizing the examination processes, routine quality control was performed daily using Attune Performance Tracking Beads and a standardized whole blood sample to verify laser alignment, detector sensitivity, fluidic stability, and antibody stability. Fluorescence intensity and coefficient of variation were compared with baseline values to ensure performance consistency. Instruments failing QC were cleaned, recalibrated, or serviced before sample acquisition.

**Data Modeling**

This study utilized immune cell detection data presented as percentages, obviating the requirement for preprocessing adjustments. The training model included 106 samples, with 70 in the non-SA-AKD group and 36 in the SA-AKD group. The RandomUnderSampler function from the Imblearn library was applied to balance the samples, after which the data were divided into training and testing sets at a 4:1 sampling strategy by using the train_test_split function of Scikit-learn. Additionally, 32 samples were obtained for validation, with 27 in the non-SA-AKD group and 5 in the SA-AKD group. To enhance the speed and accuracy of model training, min-max normalization was applied to scale the immune Gini coefficient values to a range of [0,1] without modifying the original data distribution. The normalized immune entropy values were divided into 3 sets: training (64%), validation (16%), and testing (20%) sets. A decision tree model was constructed using the DecisionTreeClassifier function of Scikit-learn (version 1.5.0) with the Gini index, which is used to assess data impurity; higher Gini values indicate greater impurity. The decision tree (DT) depth was limited to 10 layers. All models were constructed using Python, version 3.8.15.

The retrospective AKD diagnosis results were used as a reference standard. The accuracy of DT-based screening depends on whether test results indicate or exclude the target condition. The results are assigned to 4 cells in the plot for further sensitivity, specificity, and predictive value calculations. Percentages were calculated using the following formulas:

- Accuracy = (upper left + lower right)/(all) × 100
- Sensitivity = [upper left/(upper + lower left)] × 100
- Specificity = [lower right/(upper + lower right)] × 100
- False-Negative Rate (1 − Sensitivity) = lower left/(upper + lower left) × 100
- False-Positive Rate (1 − Specificity) = upper right/(upper + lower right) × 100

**Machine Learning Model Construction and Validation**

All analyses were conducted on a macOS environment using Python 3.8.15 (Clang 14.0.6). The computational workflow was built upon widely used Python libraries, including NumPy 1.24.4, Pandas 2.0.3, SciPy 1.10.1, scikit-learn 1.2.2, imbalanced-learn 0.10.0, SHAP 0.41.0, and Matplotlib 3.5.3. To address class imbalance, the RandomUnderSampler function from the imbalanced-learn package (sampling_strategy = ‘majority’) was applied before each training iteration to ensure an equal number of positive and negative samples. The dataset was then randomly divided into a training set (80%) and a testing set (20%), and model training was repeated iteratively to identify the most stable and best-performing configuration. Three supervised learning algorithms were implemented using scikit-learn, including a Decision Tree (DT), K-Nearest Neighbors (KNN), and Support Vector Machine (SVM). The DT model was constructed with the parameters criterion = ‘gini’, splitter = ‘best’, max_depth = 10, min_weight_fraction_leaf = 0.1, and random_state = 42, which effectively constrained model complexity to prevent overfitting while maintaining interpretability. The KNN model was trained using default settings, whereas the SVM model utilized an ‘rbf’ kernel for nonlinear classification. To evaluate model stability and generalizability, a 5-fold stratified cross-validation was performed exclusively on the validation cohort after model training. This procedure was used solely for model evaluation and comparison and was not part of the training process. During cross-validation, the proportion of positive and negative samples was preserved in each fold, and the mean values of performance metrics—including accuracy, sensitivity, specificity, and the area under the receiver operating characteristic curve (AUC)—were computed to assess overall model robustness and predictive reliability.

**Statistics Analysis**

An analysis of demographic characteristics and an analysis of variance were conducted across various kidney injury. Unpaired *t* tests and chi-square tests were used to assess categorical variables. Immune cell expression analyses were performed using IBM SPSS Statistics version 27, GraphPad Prism version 9.5.0, and SciPy version 1.10.0, with graphs generated in Seaborn version 0.12.2. Significance was set at *P* < 0.05.

**Tables**

**Table S1.** Patient demographic characteristics in the training and validation cohorts

| **Demographic Characteristics** | **Training (2020-2021)** | | | **Validation (2022)** | | |
| --- | --- | --- | --- | --- | --- | --- |
|  | **Non-Sepsis-Associated Acute Kidney Disease (SA-AKD) (*n* = 70)** | **SA-AKD**  ***(n* = 36)** | ***P* Value** | **Non-SA-AKD**  **(*n* = 27)** | **SA-AKD**  **(*n* = 5)** | ***P* Value** |
| Age (years) | 66 ± 18 | 67 ± 19 | .826 | 65 ± 17 | 82 ± 11 | .030 |
| **Sex** | | | | | | |
| Female (n) | 34 | 11 | .098 | 17 | 3 | 1.000 |
| Male (n) | 36 | 25 |  | 10 | 2 |  |
| Hospitalized (n) | 65 | 35 | .661 | 21 | 4 | 1.000 |
| Days in Hospital (days) | 5 ± 4 | 7 ± 5 | .589 | 4 ± 3 | 4 ± 2 | .584 |
| Sepsis Severity (n) | 11 | 6 | 1.000 | 6 | 1 | 1.000 |
| **Bacteria type** | | | | | | |
| Gram Positive Cocci (GPC) (n) | 20 | 15 | .177 | 3 | 2 | .216 |
| Gram Negative Bacteria (GNB) (n) | 47 | 18 |  | 21 | 3 |  |
| Both (n) | 2 | 3 |  | 3 | 0 |  |
| Yeast-like (n) | 1 | 0 |  | 0 | 0 |  |
| **Clinical** | | | | | | |
| Serum Urea Nitrogen (mg/dL) | 15.96 ± 14.54 | 29.59 ± 21.19 | **.000** | 16.41 ± 13.17 | 47.6 ± 36.9 | **.003** |
| Cr (mg/dL) | 0.957 ± 0.63 | 2.7 ± 6.175 | **.000** | 0.879 ± 0.456 | 1.840 ± 0.826 | **.001** |

Independent samples were evaluated using the Mann-Whitney U Test.

Sex, length of hospitalization, sepsis severity, and bacteria type were evaluated using Fischer’s exact test.

| **Demographic Characteristics** | **No AKD** | | | **AKD** | | |
| --- | --- | --- | --- | --- | --- | --- |
|  | **Training**  **(*n* = 70)** | **Validation**  **(*n* = 36)** | ***P* Value** | **Training**  **(*n* = 27)** | **Validation**  **(*n* = 5)** | ***P* Value** |
| **Clinical** | | | | | | |
| Serum Urea Nitrogen (mg/dL) | 15.96 ± 14.54 | 16.41 ± 13.17 | **.897** | 29.59 ± 21.19 | 47.6 ± 36.9 | **.175** |
| Cr (mg/dL) | 0.957 ± 0.63 | 0.879 ± 0.456 | **.684** | 2.7 ± 6.175 | 1.840 ± 0.826 | **.426** |

Independent samples were evaluated using the Mann-Whitney U Test.

**Table S2.** Demographic characteristics of all patients who did and did not experience sepsis-associated acute kidney disease (SA-AKD) progression.

| **Demographic** | **2020-2022** | | |
| --- | --- | --- | --- |
|  | **Non SA-AKD**  **(n = 97)** | **SA-AKD**  **(n = 41)** | ***P* Value** |
| Age (years old) | 66 ± 17 | 69 ± 18 | 0.3357 |
| Sex | | | |
| Female (n) | 51 | 14 | 0.0479 |
| Male (n) | 46 | 27 |  |
| Hospitalized (n) | 86 | 39 | 0.2971 |
| Day in Hospital (day) | 5 ± 4 | 7 ± 5 | 0.0514 |
| Sepsis severity (n) | 17 | 7 | 0.9494 |
| Bacteria type | | | |
| Gram Positive Cocci (GPC) (n) | 23 | 17 | 0.1001 |
| Gram Negative Bacteria (GNB) (n) | 68 | 21 |  |
| Both (n) | 5 | 3 |  |
| Yeast-like (n) | 1 | 0 |  |
| Clinical | | | |
| BUN (mg/dL) | 16.08 ± 14.03 | 31.78 ± 23.47 | <0.0001 |
| Cr (mg/dL) | 0.93 ± 0.58 | 2.59 ± 5.72 | 0.0058 |

Independent-Samples Mann-Whitney U Test

Sex, hospitalized, sepsis severity, and bacteria type >> Fischer’s exact test

**Table S3.** Expression of peripheral blood immune cell populations in patients with or without sepsis-associated acute kidney disease (SA-AKD) progression

| **Cell Population** | **Training** | | | **Validation** | | |
| --- | --- | --- | --- | --- | --- | --- |
|  | **No AKD**  **(*n* = 70)** | **AKD**  **(*n* = 36)** | ***P* Value*** | **No AKD**  **(*n* = 27)** | **AKD**  **(*n* = 5)** | ***P* Value*** |
| B | 9.53 ± 5.43 | 10.15 ± 5.42 | .674 | 7.43 ± 3.6 | 11.19 ± 3.86 | .077 |
| T | 56.01 ± 15.99 | 50.2 ± 16.1 | .084 | 49.89 ± 13.03 | 40.65 ± 11.75 | .201 |
| Th | 36.08 ± 11.79 | 32.06 ± 13.4 | .114 | 36.53 ± 9.9 | 32.97 ± 10.28 | .545 |
| Active Th(HLA-DR+) | 3.8 ± 5.54 | 5.15 ± 6.65 | .060 | 1.67 ± 1.11 | 0.96 ± 0.86 | .201 |
| **Naïve Th(CD62L+)** | 22.09 ± 11.91 | 16.07 ± 10.66 | **.009** | 26.33 ± 9.82 | 21.51 ± 8.01 | .418 |
| Naïve Th(CD45RA+) | 9.95 ± 7.77 | 7.3 ± 7.47 | .061 | 10.48 ± 6.59 | 5.17 ± 3.5 | .109 |
| Memory Th(CD62L-HLA-DR+) | 10.19 ± 7.02 | 10.85 ± 6.82 | .580 | 8.52 ± 3.2 | 10.53 ± 5.66 | .687 |
| Memory Th(CD45RO+) | 11.61 ± 9.38 | 11.76 ± 8.12 | .813 | 13.86 ± 6.8 | 18.06 ± 10.83 | .545 |
| Regulatory Th | 2.27 ± 2.82 | 1.61 ± 1.78 | .223 | 0.9 ± 0.5 | 1.09 ± 0.39 | .285 |
| Th1 | 13.48 ± 7.41 | 10.89 ± 7.09 | .069 | 11.34 ± 4.42 | 12.92 ± 7.44 | .614 |
| **Naïve Th1** | 1.2 ± 1.15 | 1.11 ± 2.09 | **.020** | 0.79 ± 0.89 | 0.54 ± 0.43 | 1.000 |
| Memory Th1 | 6.06 ± 6.15 | 5.2 ± 4.92 | .729 | 6.76 ± 3.6 | 9.96 ± 7.17 | .418 |
| Th2 | 1.16 ± 1.38 | 0.85 ± 1 | .872 | 1.63 ± 1.34 | 1.2 ± 1.2 | .545 |
| Naïve Th2 | 0.4 ± 0.63 | 0.23 ± 0.33 | .543 | 0.47 ± 0.45 | 0.24 ± 0.43 | .150 |
| Memory Th2 | 0.37 ± 0.46 | 0.36 ± 0.47 | .845 | 0.7 ± 0.71 | 0.53 ± 0.52 | .801 |
| Treg | 1.05 ± 1.58 | 0.69 ± 0.97 | .670 | 2.78 ± 1.84 | 2.48 ± 2.05 | .920 |
| Naïve Treg | 0.16 ± 0.34 | 0.04 ± 0.09 | .050 | 0.48 ± 0.53 | 0.24 ± 0.48 | .389 |
| Memory Treg | 0.46 ± 0.81 | 0.33 ± 0.52 | .592 | 1.05 ± 0.95 | 1.02 ± 0.83 | .960 |
| Th17 | 1.74 ± 3 | 1.24 ± 1.55 | .832 | 3.19 ± 2.68 | 4.11 ± 3.83 | .725 |
| Naïve Th17 | 1.74 ± 3 | 1.24 ± 1.55 | .832 | 3.19 ± 2.68 | 4.11 ± 3.83 | .725 |
| Memory Th17 | 0.91 ± 1.8 | 0.71 ± 1.16 | .623 | 2.03 ± 1.79 | 2.76 ± 3.78 | .763 |
| Th22 | 1.72 ± 3.2 | 1.72 ± 2.92 | .625 | 0.71 ± 1.25 | 0.69 ± 0.8 | .650 |
| Naïve Th22 | 0.09 ± 0.25 | 0.07 ± 0.18 | .935 | 0.02 ± 0.09 | 0 ± 0 | .801 |
| Memory Th22 | 0.88 ± 1.83 | 1.07 ± 1.9 | .492 | 0.18 ± 0.32 | 0.1 ± 0.14 | .920 |
| Tfh | 0.41 ± 0.7 | 0.93 ± 2.2 | .558 | 0.65 ± 0.82 | 0.64 ± 0.63 | 1.000 |
| Naïve Tfh | 0.06 ± 0.12 | 0.11 ± 0.43 | .807 | 0.05 ± 0.07 | 0.04 ± 0.09 | .650 |
| Memory Tfh | 0.15 ± 0.24 | 0.4 ± 0.95 | .699 | 0.44 ± 0.59 | 0.46 ± 0.44 | .841 |
| Tc | 15.46 ± 10.62 | 13.72 ± 8.99 | .443 | 9.78 ± 8.07 | 4.7 ± 3.26 | .109 |
| Active Tc | 0.08 ± 0.16 | 0.45 ± 1.65 | .794 | 0.09 ± 0.16 | 0.05 ± 0.09 | .725 |
| Naïve Tc | 4.78 ± 5.95 | 3.3 ± 2.28 | .513 | 5.18 ± 5.46 | 1.6 ± 0.85 | .077 |
| Memory Tc | 10.49 ± 7.41 | 9.86 ± 8.43 | .443 | 4.42 ± 4.83 | 3.01 ± 2.64 | .511 |
| **Regulatory Tc** | 3.02 ± 5.38 | 1.76 ± 2.64 | .319 | 0.52 ± 0.88 | 0.08 ± 0.08 | **.004** |
| DP T | 0.69 ± 0.64 | 0.73 ± 0.74 | .844 | 0.29 ± 0.38 | 0.17 ± 0.07 | .880 |
| DN T | 3.78 ± 2.37 | 3.71 ± 2.29 | .805 | 3.3 ± 1.7 | 2.79 ± 0.59 | .725 |
| NK | 14.16 ± 8.2 | 17.09 ± 11.47 | .257 | 18.59 ± 8.82 | 23.42 ± 14.07 | .448 |
| CD56b NK | 0.13 ± 0.29 | 0.17 ± 0.42 | .991 | 0.23 ± 0.29 | 0.12 ± 0.27 | .241 |
| CD56d NK | 10.54 ± 6.8 | 11.49 ± 11.42 | .577 | 14.19 ± 8.54 | 16.8 ± 15.28 | .960 |
| **CD56b** | 0.13 ± 0.18 | 0.07 ± 0.13 | **.049** | 0.26 ± 0.24 | 0.37 ± 0.29 | .361 |
| **CD56d** | 3.36 ± 3.77 | 5.36 ± 4.54 | **.004** | 3.91 ± 2.86 | 6.13 ± 4.85 | .220 |
| NKT | 8.92 ± 8.13 | 7.93 ± 7.83 | .340 | 12.28 ± 7.61 | 7.87 ± 1.06 | .285 |
| NKT CD8 | 5.4 ± 6.28 | 4.97 ± 6.44 | .366 | 9.16 ± 6.37 | 6.31 ± 1.18 | .579 |
| NKT CD4 | 1.84 ± 2.02 | 1.43 ± 1.25 | .400 | 1.73 ± 1.94 | 0.83 ± 0.98 | .201 |
| NKT DP | 0.41 ± 0.46 | 0.51 ± 0.6 | .771 | 0.42 ± 0.39 | 0.34 ± 0.26 | .841 |
| NKT DN | 1.26 ± 1.4 | 1.03 ± 1.02 | .602 | 0.98 ± 1.85 | 0.38 ± 0.25 | .220 |
| CD56b NKT | 0.04 ± 0.11 | 0.03 ± 0.06 | .261 | 0.07 ± 0.06 | 0.04 ± 0.05 | .361 |
| CD56d NKT | 0.87 ± 1.3 | 0.85 ± 1.22 | .476 | 0.78 ± 1.82 | 0.4 ± 0.5 | .448 |
| CD56b | 0.18 ± 0.23 | 0.12 ± 0.16 | .286 | 0.44 ± 0.38 | 0.39 ± 0.13 | .763 |
| CD56d | 7.83 ± 7.45 | 6.93 ± 7.23 | .248 | 10.97 ± 7.36 | 7.04 ± 0.87 | .545 |
| DC | 3.63 ± 4.92 | 4.37 ± 5.72 | .573 | 3.96 ± 3.23 | 4.35 ± 1.72 | .361 |
| Mature DC | 1.43 ± 2.96 | 1.25 ± 2.33 | .912 | 1.3 ± 1.49 | 0.94 ± 0.75 | .880 |
| Immature DC | 2.2 ± 2.73 | 3.12 ± 4 | .479 | 2.65 ± 2.04 | 3.36 ± 1.21 | .241 |
| Monocyte | 7.74 ± 8.29 | 10.25 ± 10.13 | .236 | 7.85 ± 6.42 | 12.52 ± 9.73 | .241 |
| C. Monocyte | 6.52 ± 7.65 | 8.76 ± 9.41 | .230 | 6.08 ± 5.58 | 9.59 ± 8.92 | .418 |
| NC. Monocyte | 0.85 ± 1.16 | 1.01 ± 1.97 | .957 | 1.22 ± 1.79 | 1.56 ± 1.47 | .310 |
| Inter. Monocyte | 0.37 ± 0.62 | 0.48 ± 0.84 | .874 | 0.55 ± 0.91 | 1.37 ± 1.72 | .068 |

*After applying the Benjamini–Hochberg false discovery rate (FDR) correction with a significance threshold of q < 0.2, four immune cell subsets remained statistically significant across the training and validation cohorts. In the training set, CD56d NK cells (p = 0.004, q = 0.084), naïve Th (CD62L⁺) cells (p = 0.009, q = 0.162), and naïve Th1 cells (p = 0.020, q = 0.176) showed significant differences between patients with and without AKD. In the validation set, regulatory Tc cells (p = 0.004, q = 0.172) remained significant after adjustment.

**Table S4** Expression of peripheral blood immune cell populations of patients who did and did not experience sepsis-associated acute kidney disease (SA-AKD) progression

| **Cell Population** | **Total** | | |
| --- | --- | --- | --- |
|  | **No AKD (n = 97)** | **AKD (n = 41)** | ***P* Value*** |
| B | 8.94% ± 0.05 | 10.27% ± 0.05 | 0.1653 |
| T | 54.31% ± 0.15 | 49.04% ± 0.16 | 0.0708 |
| Th | 36.21% ± 0.11 | 32.18% ± 0.13 | 0.0683 |
| Active Th(HLADR+) | 3.21% ± 0.05 | 4.63% ± 0.06 | 0.1525 |
| Naïve Th (CD62L+) | 23.27% ± 0.11 | 16.73% ± 0.10 | 0.0021 |
| Naïve Th(CD45RA+) | 10.10% ± 0.07 | 7.04% ± 0.07 | 0.0267 |
| Memory Th(CD62L-HLADR+) | 9.73% ± 0.06 | 10.81% ± 0.07 | 0.3601 |
| Memory Th(CD45RO+) | 12.24% ± 0.09 | 12.53% ± 0.08 | 0.8552 |
| Regulatory Th | 1.88% ± 0.02 | 1.54% ± 0.02 | 0.423 |
| Th1 | 12.89% ± 0.07 | 11.13% ± 0.07 | 0.1723 |
| Naïve Th1 | 1.08% ± 0.01 | 1.04% ± 0.02 | 0.8675 |
| Memory Th1 | 6.26% ± 0.06 | 5.78% ± 0.05 | 0.6398 |
| Th2 | 1.29% ± 0.01 | 0.89% ± 0.01 | 0.1015 |
| Naïve Th2 | 0.42% ± 0.01 | 0.23% ± 0.00 | 0.0539 |
| Memory Th2 | 0.46% ± 0.01 | 0.38% ± 0.00 | 0.4306 |
| Treg | 1.53% ± 0.02 | 0.91% ± 0.01 | 0.0461 |
| Naïve Treg | 0.25% ± 0.00 | 0.07% ± 0.00 | 0.0073 |
| Memory Treg | 0.63% ± 0.01 | 0.42% ± 0.01 | 0.1661 |
| Th17 | 2.15% ± 0.03 | 1.59% ± 0.02 | 0.2786 |
| Naïve Th17 | 2.15% ± 0.03 | 1.59% ± 0.02 | 0.2786 |
| Memory Th17 | 1.22% ± 0.02 | 0.96% ± 0.02 | 0.4561 |
| Th22 | 1.44% ± 0.03 | 1.60% ± 0.03 | 0.7585 |
| Naïve Th22 | 0.07% ± 0.00 | 0.06% ± 0.00 | 0.9104 |
| Memory Th22 | 0.68% ± 0.02 | 0.96% ± 0.02 | 0.3794 |
| Tfh | 0.48% ± 0.01 | 0.89% ± 0.02 | 0.0829 |
| Naïve Tfh | 0.06% ± 0.00 | 0.10% ± 0.00 | 0.3282 |
| Memory Tfh | 0.23% ± 0.00 | 0.41% ± 0.01 | 0.1049 |
| Tc | 13.88% ± 0.10 | 12.62% ± 0.09 | 0.4946 |
| Active Tc | 0.09% ± 0.00 | 0.40% ± 0.02 | 0.0512 |
| Naïve Tc | 4.90% ± 0.06 | 3.10% ± 0.02 | 0.0561 |
| Memory Tc | 8.80% ± 0.07 | 9.03% ± 0.08 | 0.8715 |
| Regulatory Tc | 2.32% ± 0.05 | 1.56% ± 0.02 | 0.329 |
| DP T | 0.58% ± 0.01 | 0.66% ± 0.01 | 0.4726 |
| DN T | 3.65% ± 0.02 | 3.60% ± 0.02 | 0.8994 |
| NK | 15.40% ± 0.09 | 17.86% ± 0.12 | 0.1718 |
| CD56b NK | 0.16% ± 0.00 | 0.16% ± 0.00 | 0.971 |
| CD56d NK | 11.56% ± 0.07 | 12.13% ± 0.12 | 0.7318 |
| CD56b | 0.17% ± 0.00 | 0.11% ± 0.00 | 0.1046 |
| CD56d | 3.51% ± 0.04 | 5.46% ± 0.04 | 0.0076 |
| NKT | 9.85% ± 0.08 | 7.92% ± 0.07 | 0.1902 |
| NKT CD8 | 6.45% ± 0.06 | 5.13% ± 0.06 | 0.2702 |
| NKT CD4 | 1.81% ± 0.02 | 1.36% ± 0.01 | 0.1775 |
| NKT DP | 0.41% ± 0.00 | 0.49% ± 0.01 | 0.411 |
| NKT DN | 1.18% ± 0.02 | 0.95% ± 0.01 | 0.3862 |
| CD56b NKT | 0.05% ± 0.00 | 0.04% ± 0.00 | 0.5073 |
| CD56d NKT | 0.84% ± 0.01 | 0.79% ± 0.01 | 0.8476 |
| CD56b | 0.25% ± 0.00 | 0.15% ± 0.00 | 0.0554 |
| CD56d | 8.71% ± 0.07 | 6.94% ± 0.07 | 0.1963 |
| DC | 3.72% ± 0.04 | 4.36% ± 0.05 | 0.4723 |
| Mature DC | 1.39% ± 0.03 | 1.21% ± 0.02 | 0.7014 |
| Inmature DC | 2.33% ± 0.03 | 3.15% ± 0.04 | 0.1391 |
| Monocyte | 7.77% ± 0.08 | 10.53% ± 0.10 | 0.0835 |
| C. Monocyte | 6.39% ± 0.07 | 8.86% ± 0.09 | 0.0922 |
| NC. Monocyte | 0.95% ± 0.01 | 1.08% ± 0.02 | 0.6685 |
| Inter. Monocyte | 0.42% ± 0.01 | 0.59% ± 0.01 | 0.281 |

*After correction for multiple comparisons using the Benjamini–Hochberg false discovery rate (FDR) procedure with a significance threshold of q < 0.2, four immune cell subsets remained significant. Naïve Th (CD62L⁺) cells (p = 0.0021, q = 0.09), CD56d NK cells (p = 0.0076, q = 0.128), naïve Treg cells (p = 0.0073, q = 0.128), and naïve Th (CD45RA⁺) cells (p = 0.0267, q = 0.171) showed significant differences between AKD and non-AKD groups. All other immune subsets had FDR-adjusted q values ≥ 0.242 and were not statistically significant.

**Table S5.** Performance metrics of the composite decision tree (DT) model integrating renal indices (BUN, Cr) and immune cell subsets (naïve Treg, CD56^dim NK cells). The model demonstrated high predictive accuracy and balanced discrimination, with an overall accuracy of 88.7%, sensitivity of 94.4%, specificity of 85.7%, PPV of 77.3%, NPV of 96.8%, F1-score of 85.0%, and AUC of 0.91.

| **Accuracy** | **Sensitivity** | **Specificity** | **PPV** | **NPV** | **F1-score** | **AUC** |
| --- | --- | --- | --- | --- | --- | --- |
| 88.7% | 94.4% | 85.7% | 77.3% | 96.8% | 85% | 91% |

**Table S6.** Five-fold cross-validation performance of the SA-AKD classification model after class balancing (SA-Non = 41, SA-AKD = 41). The model achieved stable predictive performance across folds, with an average accuracy of 79.3%, sensitivity of 82.8%, and specificity of 83.1%.

|  | 1 | 2 | 3 | 4 | 5 | Mean |
| --- | --- | --- | --- | --- | --- | --- |
| Accuracy | 70.6% | 82.4% | 87.5% | 75.0% | 81.3% | 79.3% |
| Sensitivity | 75.0% | 88.9% | 87.5% | 87.5% | 75.0% | 82.8% |
| Specificity | 77.8% | 87.5% | 87.5% | 62.5% | 100.0% | 83.1% |

**Table S7.** Antibody panel composition.

| **Panel#1** | | | | |
| --- | --- | --- | --- | --- |
| **Antibody** | **Fluorochrome** | **Source** | **Catalog Number** |  |
| CD3 | eFluor 450 | Thermo Fisher | 48-0037-42 |  |
| CD19 | eFluor506 | Thermo Fisher | 69-0199-42 |  |
| CD56 | Super Bright 600 | Thermo Fisher | 63-0566-42 |  |
| CD4 | PerCP | BioLegend | 317432 |  |
| CD8 | PE-Cyanine 7 | Thermo Fisher | 25-0086-42 |  |
| CD11c | PE | BioLegend | 301606 |  |
| CD14 | APC | Thermo Fisher | 17-0149-42 |  |
| CD16 | Alexa Fluor 488 | BioLegend | 302019 |  |
| CD25 | APC-eFluor 780 | Thermo Fisher | 47-0257-42 |  |
| HLA-DR | Brilliant Violet 711 | BioLegend | 307644 |  |
| CD62L | Alexa Fluor 700 | BioLegend | 304820 |  |
| **Panel#2** | | | | |
| **Antibody** | **Fluorochrome** | **Source** | **Catalog Number** |  |
| CD3 | eFluor 450 | Thermo Fisher | 48-0037-42 |  |
| CD4 | eFluor506 | Thermo Fisher | 69-0049-42 |  |
| CXCR3 | PE-Cyanine 7 | Thermo Fisher | 25-1839-42 |  |
| CCR3 | APC | Thermo Fisher | 17-1939-42 |  |
| CCR6 | FITC | Thermo Fisher | 11-1969-42 |  |
| CCR10 | PE | BioLegend | 341504 |  |
| CXCR5 | Alexa Fluor 700 | BioLegend | 356916 |  |
| CCR5 | PerCP-eFluor710 | Thermo Fisher | 46-1956-42 |  |
| CD25 | APC-eFluor 780 | Thermo Fisher | 47-0257-42 |  |
| CD45RA | Super Bright 600 | Thermo Fisher | 63-0458-42 |  |
| CD45RO | Super Bright 702 | Thermo Fisher | 67-0457-42 |  |

**Figures**

**Fig. S1** Comparison of clinical parameters in patients with or without sepsis-associated acute kidney disease (SA-AKD) progression. **A** Classification of patients with or without SA-AKD progression. **B** Expression of serum blood urea nitrogen (BUN) and creatinine (Cr) levels in SA-AKD. **C** Expression of circulating leukocytes in SA-AKD. Significant differences were assessed using the independent-samples Kruskal-Wallis test. ^∗^*P* < .05, ^∗∗^*P* < .01, and ^∗∗∗^*P* < .001. ns, not significant.

**
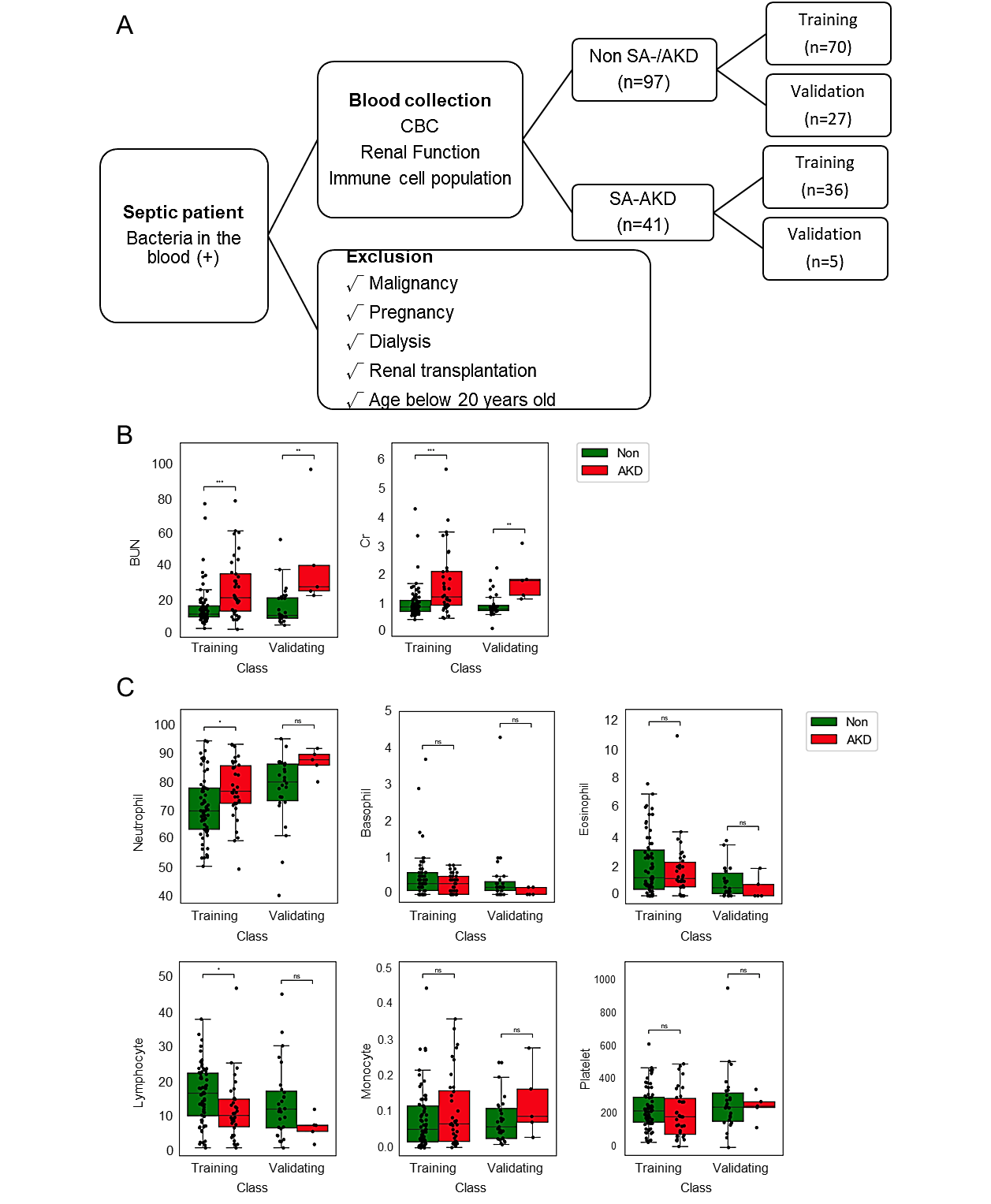
**

**Fig. S2** Comparison of clinical parameters in patients who did and did not experience sepsis-associated acute kidney disease (SA-AKD) progression. **A** Expression of serum blood urea nitrogen (BUN) and creatinine (Cr) in patients with SA-AKD. **B** Expression of circulating leukocytes in patients with SA-AKD. ^∗^*P* < .05, ^∗∗^*P* < .01, and ^∗∗∗∗^*P* < .0001. ns, not significant.


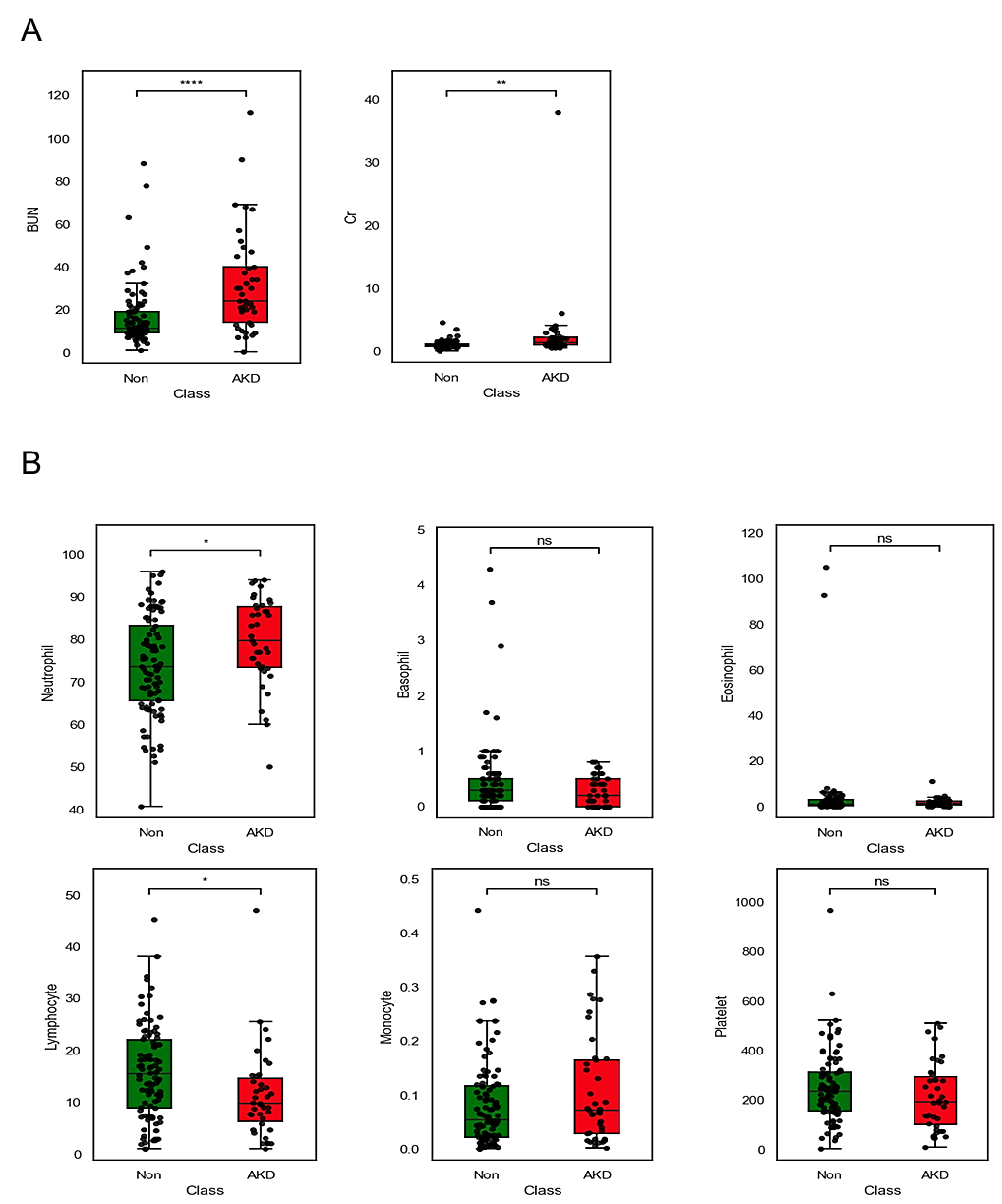


**Fig. S3** Immune cell expression in patients with or without sepsis-associated acute kidney disease (SA-AKD) progression. **A** Gating strategy for immune cell analysis using high-parameter flow cytometry. **B** Heat map of immune cell expression. **C** Significant changes in immune cell expression in patients with or without SA-AKD progression. Significant differences were assessed using the independent-samples Kruskal-Wallis test. ^∗^*P* < .05 and ^∗∗^*P* < .01. ns, not significant. **D** Accuracy of SA-AKD prediction made using principal component analysis (PCA). **E** Sensitivity and specificity of SA-AKD prediction made using principal component analysis.


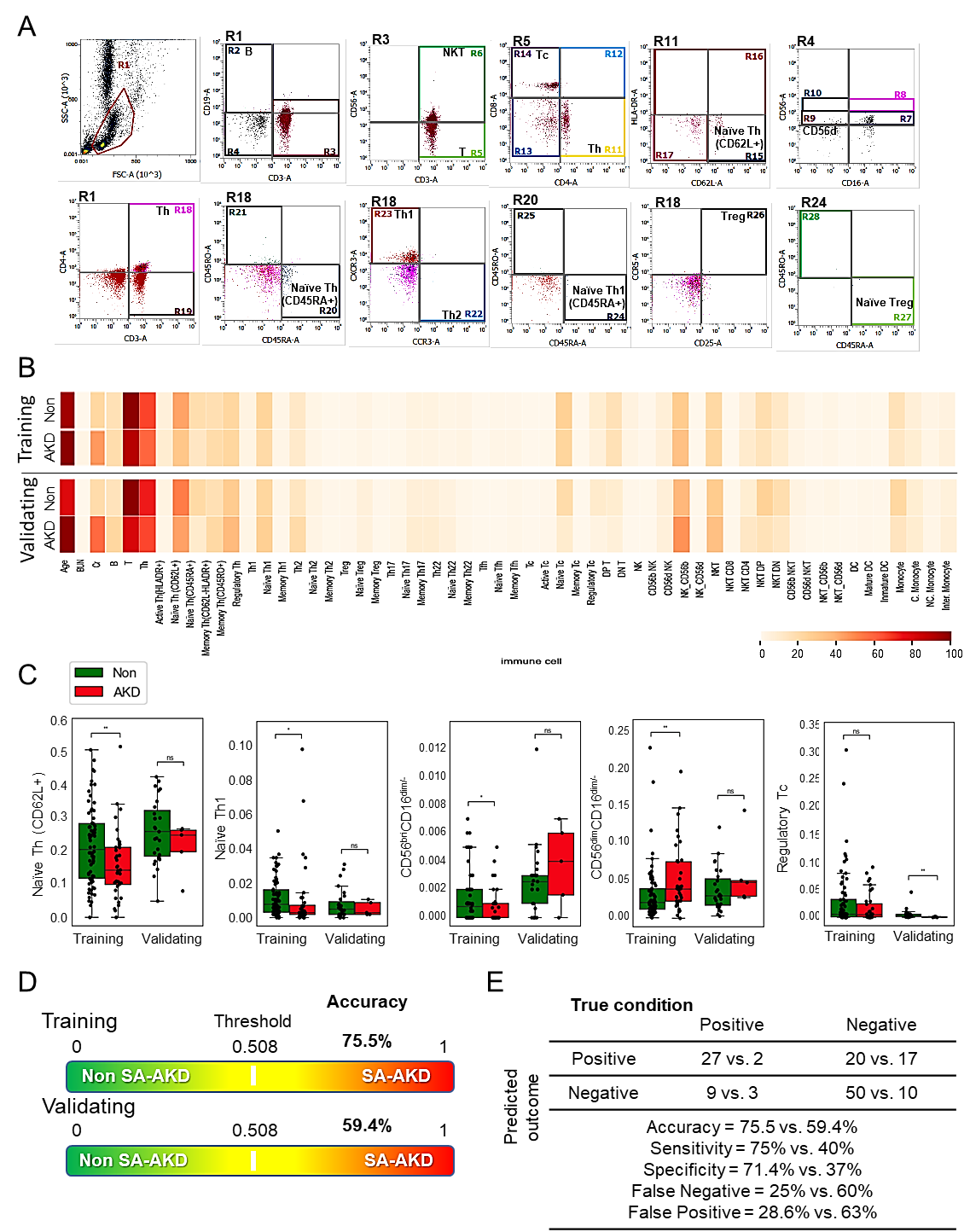

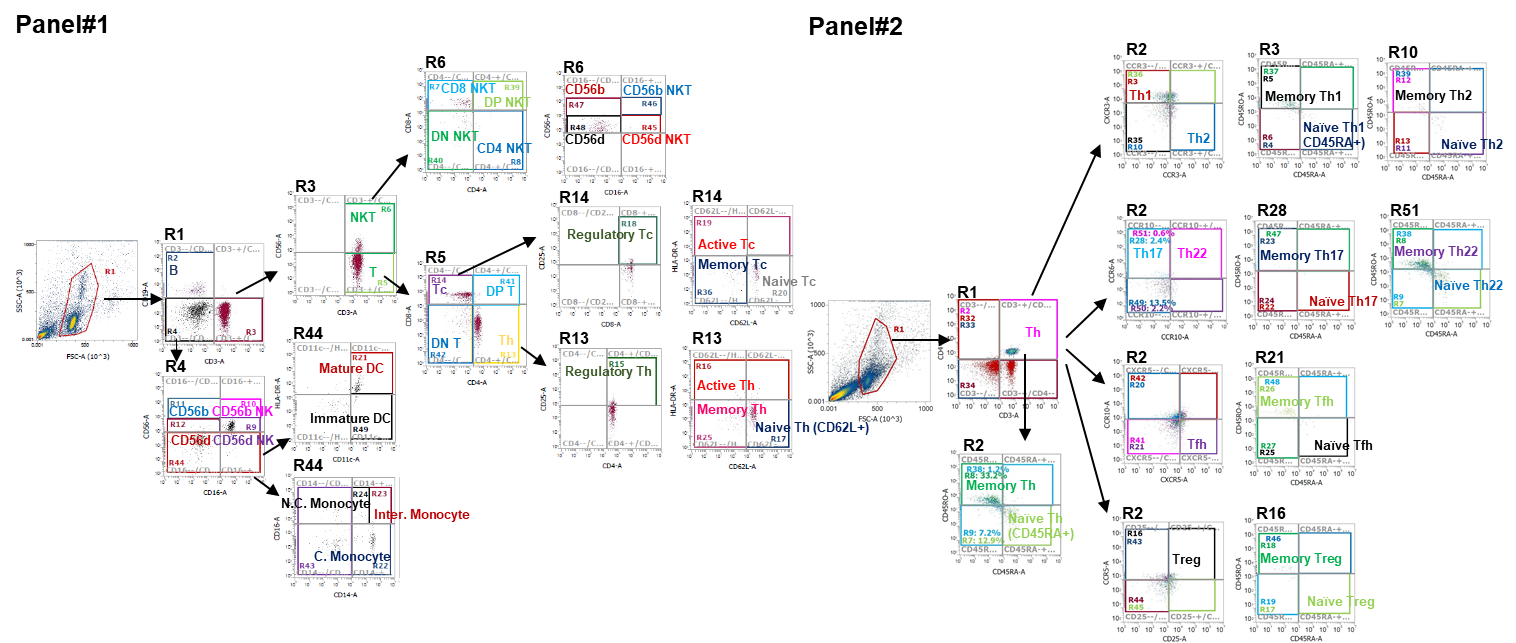


**Fig. S4** Immune cell expression in patients who did and did not experience sepsis-associated acute kidney disease (SA-AKD) progression. **A** Heat map of immune cell expression. **B** Significant changes in immune cell expression in patients with or without SA-AKD progression. **C** Accuracy of SA-AKD predictions made using principal component analysis (PCA). **D** Sensitivity and specificity of SA-AKD predictions made using PCA.

**
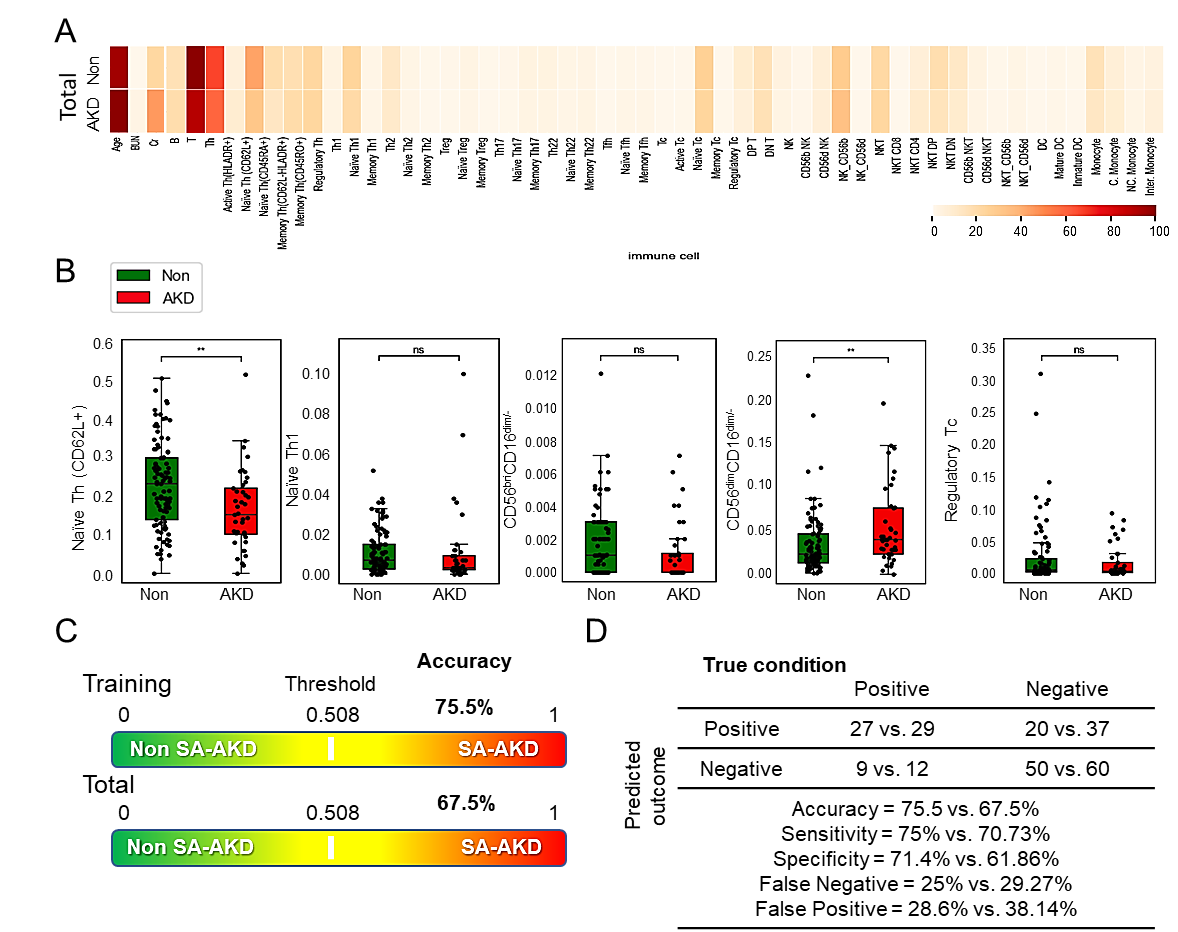
**

**Fig. S5** Decision tree (DT)-based prediction made using immune cell populations to evaluate the risk of sepsis-associated acute kidney disease (SA-AKD) progression. **A** Accuracy of SA-AKD predictions made using DT. **B** Sensitivity, specificity, and area under the receiver operating characteristic curve (AUC) of SA-AKD predictions made using DT.

**
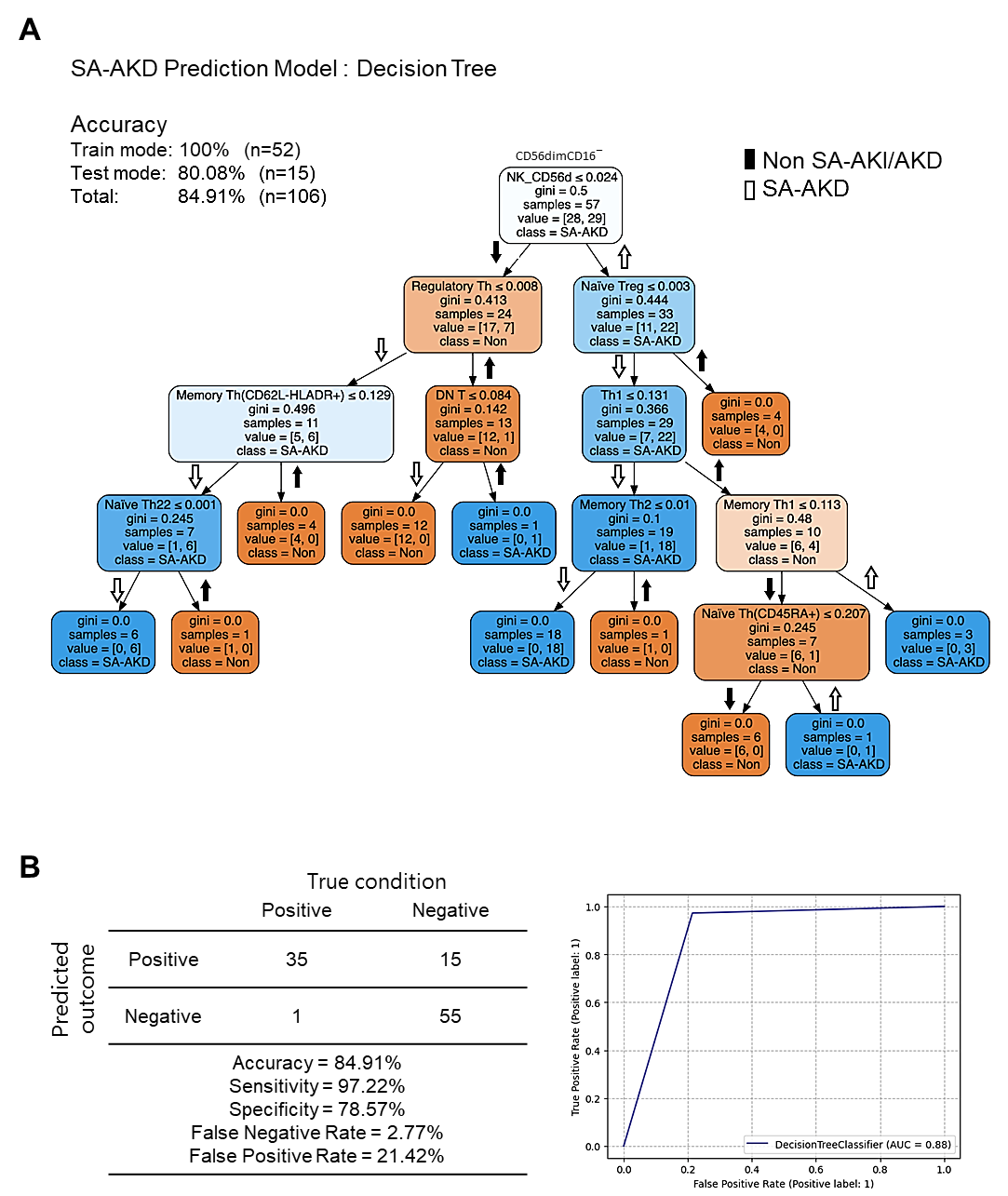
**

**Fig.S6** Decision tree (DT)-based predictions made using immune cell populations and clinical parameters for evaluating the risks of sepsis-associated acute kidney disease (SA-AKD) progression: validation sample. **A** Accuracy, sensitivity, specificity, and area under the receiver operating characteristic curve (AUC) of DT-based SA-AKD predictions made using immune cell populations. **B** Accuracy, sensitivity, specificity, and AUC of DT-based SA-AKD predictions made using clinical parameters.


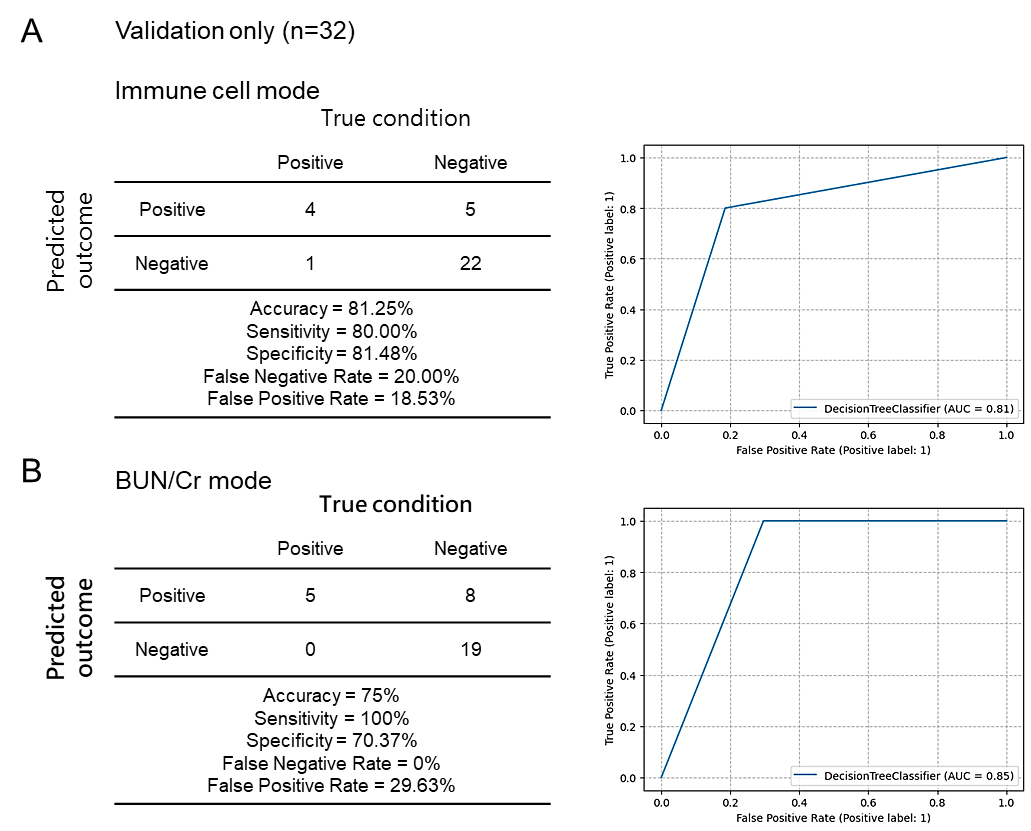


**Fig. S7** Decision tree (DT)-based prediction made using clinical parameters to evaluate the risk of sepsis-associated acute kidney disease (SA-AKD) progression. **A** Accuracy of SA-AKD predictions made using DT. **B** Sensitivity, specificity, and area under the receiver operating characteristic curve (AUC) of SA-AKD predictions made using DT.

**
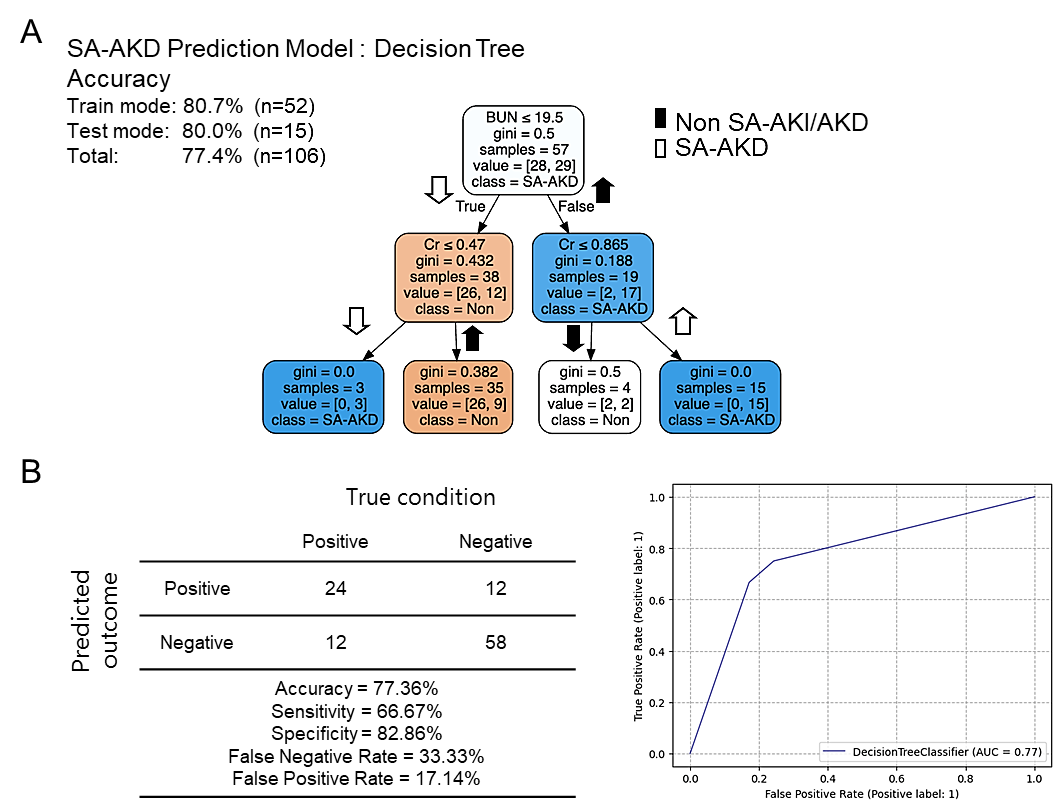
**

**Fig. S8.** Decision tree (DT)-based predictions made using all parameters to evaluate the risk of sepsis-associated acute kidney disease (SA-AKD) progression. **A** Accuracy of SA-AKD prediction made using DT. **B** Sensitivity, specificity, and area under the receiver operating characteristic curve (AUC) of SA-AKD predictions made using DT.

**
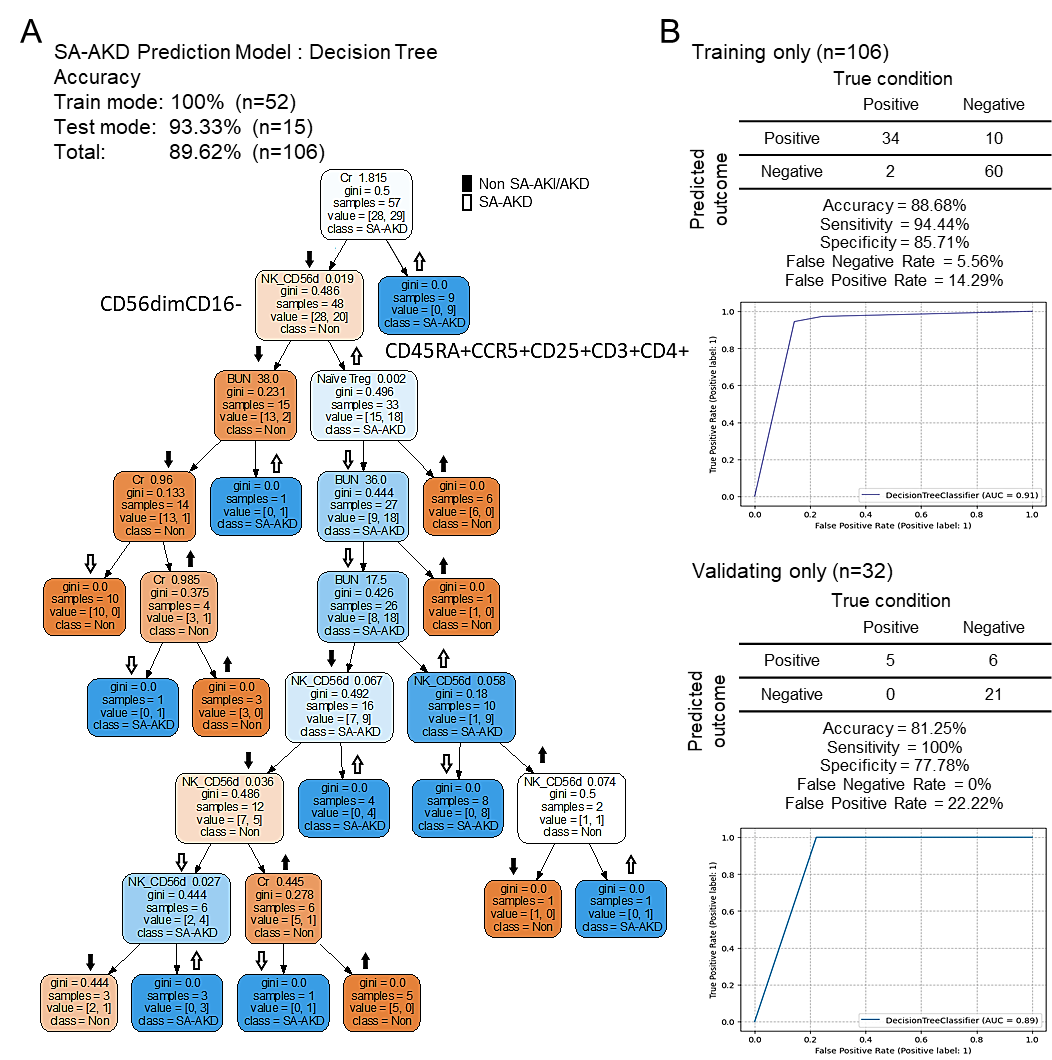
**

**Fig. S9** Immune cell populations correlated with renal dysfunction. **A** On the basis of the results of decision tree (DT)-based predictions, populations of naïve Treg cells and CD56^dim^ NK cells were measured in patients with non-sepsis-associated-acute kidney disease (SA-AKD) and SA-AKD. **B** Correlation of the expression of these cells with renal function parameters, specifically with serum blood urea nitrogen (BUN) and creatinine (Cr) levels. Significant differences were assessed using the independent-samples Kruskal-Wallis test. ^∗∗^*P* < .01. ns, not significant.

**
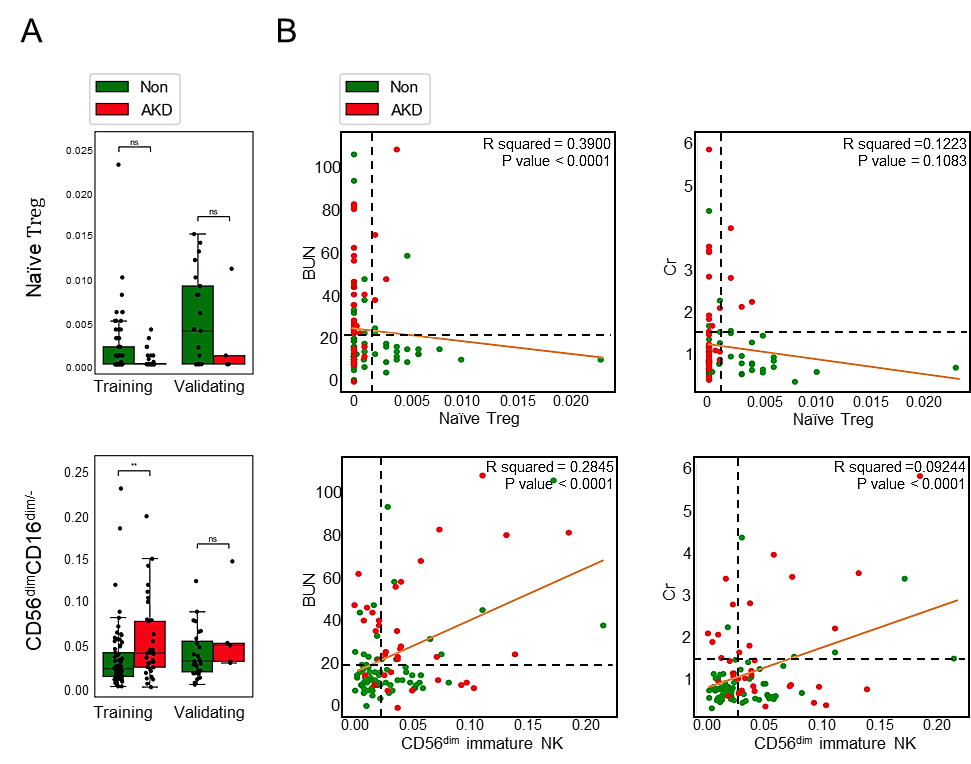
**

**Fig. S10** SHAP summary plot showing the top 10 features contributing to SA-AKD classification. Each dot represents a single sample, with color indicating the normalized feature value (red = high, blue = low). Features such as BUN, creatinine (Cr), and NK_CD56d exhibited the highest SHAP values, indicating their strong influence on the model’s prediction outcome.


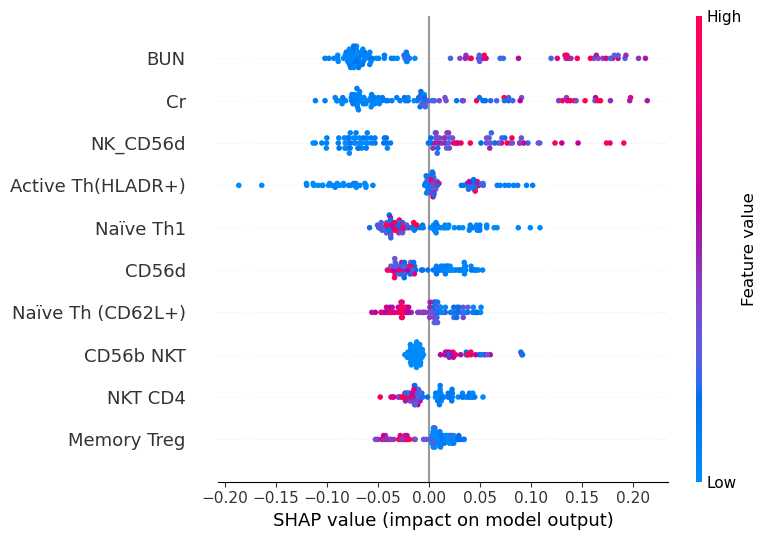

Supplement: Supplementary file 1 — Supplementary Material 1 [file 40364_2025_870_MOESM1_ESM.docx]
